# Neurodevelopmental disorders in children aged 2–9 years: Population-based burden estimates across five regions in India

**DOI:** 10.1371/journal.pmed.1002615

**Published:** 2018-07-24

**Authors:** Narendra K. Arora, M. K. C. Nair, Sheffali Gulati, Vaishali Deshmukh, Archisman Mohapatra, Devendra Mishra, Vikram Patel, Ravindra M. Pandey, Bhagabati C. Das, Gauri Divan, G. V. S. Murthy, Thakur D. Sharma, Savita Sapra, Satinder Aneja, Monica Juneja, Sunanda K. Reddy, Praveen Suman, Sharmila B. Mukherjee, Rajib Dasgupta, Poma Tudu, Manoja K. Das, Vinod K. Bhutani, Maureen S. Durkin, Jennifer Pinto-Martin, Donald H. Silberberg, Rajesh Sagar, Faruqueuddin Ahmed, Nandita Babu, Sandeep Bavdekar, Vijay Chandra, Zia Chaudhuri, Tanuj Dada, Rashna Dass, M. Gourie-Devi, S. Remadevi, Jagdish C. Gupta, Kumud K. Handa, Veena Kalra, Sunil Karande, Ramesh Konanki, Madhuri Kulkarni, Rashmi Kumar, Arti Maria, Muneer A. Masoodi, Manju Mehta, Santosh Kumar Mohanty, Harikumaran Nair, Poonam Natarajan, A. K. Niswade, Atul Prasad, Sanjay K. Rai, Paul S. S. Russell, Rohit Saxena, Shobha Sharma, Arun K. Singh, Gautam B. Singh, Leena Sumaraj, Saradha Suresh, Alok Thakar, Sujatha Parthasarathy, Bhadresh Vyas, Ansuman Panigrahi, Munish K. Saroch, Rajan Shukla, K. V. Raghava Rao, Maria P. Silveira, Samiksha Singh, Vivek Vajaratkar

**Affiliations:** 1 The INCLEN Trust International, New Delhi, India; 2 Kerala University of Health Sciences, Medical College PO, Thrissur, Kerala, India; 3 Department of Paediatrics, All India Institute of Medical Sciences, New Delhi, India; 4 Department of Paediatrics, Maulana Azad Medical College, New Delhi, India; 5 Sangath, Bardez, Goa, India; 6 Department of Global Health and Social Medicine, Harvard Medical School, Boston, Massachusetts, United States of America; 7 Department of Biostatistics, All India Institute of Medical Sciences, New Delhi, India; 8 Department of Community Medicine, Kalinga Institute of Medical Sciences, Bhubaneshwar, Odisha, India; 9 Indian Institute of Public Health, Hyderabad, Telangana, India; 10 Himachal Foundation, Dharamshala, Kangra, Himachal Pradesh, India; 11 Department of Paediatrics, Lady Hardinge Medical College, New Delhi, India; 12 Centre for Applied Research and Education on Neurodevelopmental Impairments and Disability related Health Initiatives (CARENIDHI), New Delhi, India; 13 Department of Paediatrics, Sir Ganga Ram Hospital, New Delhi, India; 14 Department of Social Medicine and Community Health, Jawaharlal Nehru University, New Delhi, India; 15 Department of Paediatrics, Stanford University School of Medicine and Lucile Packard Children’s Hospital, California, United States of America; 16 Department of Population Health Sciences and Paediatrics, and Waisman Center, University of Wisconsin School of Medicine and Public Health, Madison, Wisconsin, United States of America; 17 University of Pennsylvania School of Nursing and School of Medicine, Philadelphia, United States of America; 18 Department of Neurology, Perelman School of Medicine, University of Pennsylvania, Philadelphia, United States of America; 19 Department of Psychiatry, All India Institute of Medical Science, New Delhi, India; 20 Integral Institute of Medical Sciences & Research, Integral University, Lucknow, Uttar Pradesh, India; 21 Department of Psychology, Delhi University, New Delhi, India; 22 Department of Paediatrics, Seth GS Medical College & KEM Hospital, Mumbai, Maharashtra, India; 23 Department of Neurology, Paras Hospital, Gurugram, Haryana, India; 24 Department of Ophthalmology, Lady Hardinge Medical College, New Delhi, India; 25 Dr Rajendra Prasad Centre for Ophthalmic Sciences, All India Institute of Medical Sciences, New Delhi, India; 26 Department of Paediatric Disciplines, Health City Hospital, Guwahati, Assam, India; 27 Department of Neurology, Institute of Human Behaviour and Allied Sciences & Department of Neurophysiology, Sir Ganga Ram Hospital, New Delhi, India; 28 School of Health Policy and Planning, Kerala University of Health Sciences, Thiruvananthapuram, Kerala, India; 29 Ali Yavar Jung National Institute of Speech and Hearing Disabilities, Department of Empowerment of Persons with Disabilities, Kasturba Niketan, New Delhi, India; 30 Department of ENT & Head Neck Surgery, Medanta Medicity, Gurugram, Haryana, India; 31 Department of Paediatrics, Indraprastha Apollo Hospital, New Delhi, India; 32 Department of Paediatric Neurology, Rainbow Children’s Hospital, Hyderabad, Telengana, India; 33 Department of Paediatrics, Mumbai Port Trust Hospital, Mumbai, Maharashtra, India; 34 Department of Paediatrics, King George Medical University, Lucknow, Uttar Pradesh, India; 35 Department of Neonatology, Post Graduate Institute of Medical Education and Research and Dr. Ram Manohar Lohia Hospital, Delhi, India; 36 Department of Community Medicine, Government Medical College, Srinagar, Kashmir, India; 37 National Trust, Department of Empowerment of Persons with Disabilities, Ministry of Social Justice & Empowerment, Government of India, Delhi, India; 38 Vidya Sagar (formerly The Spastics Society of India), Chennai, Tamil Nadu, India; 39 Department of Paediatrics, Government Medical College, Nagpur, Maharashtra, India; 40 Social Welfare Department, Government of Bihar, Patna, India; 41 Department of Community Medicine, All India Institute of Medical Sciences, Ansari Nagar, New Delhi, India; 42 Department of Child & Adolescent Psychiatry and Facility for Children with Intellectual Disability, Christian Medical College, Vellore, Tamil Nadu, India; 43 Rashtriya Bal Swasthya Karyakram, Ministry of Health and Family Welfare, Nirman Bhawan, New Delhi, India; 44 Department of Otorhinolaryngology and Head and Neck Surgery (ENT), Lady Hardinge Medical College, New Delhi, India; 45 Child Development Centre, Medical College Campus, Thiruvananthapuram, Kerala, India; 46 Samarth, Chennai, Tamil Nadu, India; 47 Department of Otolaryngology & Head-Neck Surgery, All India Institute of Medical Sciences, New Delhi, India; 48 Department of Pediatric Neurology, The Hospital for Sick Children (SickKids), The Peter Gilgan Centre for Research and Learning, Toronto, Ontario, Canada; 49 Department of Paediatrics, M.P. Shah Government Medical College & G.G. Hospital, Jamnagar, Gujarat, India; 50 Department of ENT, Dr. Rajender Prasad Government Medical College, Kangra, Himachal Pradesh, India; 51 RVM Institute of Medical Sciences and Research Center, Laxmakkapally, Telangana, India; 52 Department of Paediatrics, Goa Medical College, Bambolim, Goa, India; 53 Department of Orthopedic Surgery, Goa Medical College, Bambolim, Goa, India; London School of Hygiene and Tropical Medicine, UNITED KINGDOM

## Abstract

**Background:**

Neurodevelopmental disorders (NDDs) compromise the development and attainment of full social and economic potential at individual, family, community, and country levels. Paucity of data on NDDs slows down policy and programmatic action in most developing countries despite perceived high burden.

**Methods and findings:**

We assessed 3,964 children (with almost equal number of boys and girls distributed in 2–<6 and 6–9 year age categories) identified from five geographically diverse populations in India using cluster sampling technique (probability proportionate to population size). These were from the North-Central, i.e., Palwal (*N* = 998; all rural, 16.4% non-Hindu, 25.3% from scheduled caste/tribe [SC-ST] [these are considered underserved communities who are eligible for affirmative action]); North, i.e., Kangra (*N* = 997; 91.6% rural, 3.7% non-Hindu, 25.3% SC-ST); East, i.e., Dhenkanal (*N* = 981; 89.8% rural, 1.2% non-Hindu, 38.0% SC-ST); South, i.e., Hyderabad (*N* = 495; all urban, 25.7% non-Hindu, 27.3% SC-ST) and West, i.e., North Goa (*N* = 493; 68.0% rural, 11.4% non-Hindu, 18.5% SC-ST). All children were assessed for vision impairment (VI), epilepsy (Epi), neuromotor impairments including cerebral palsy (NMI-CP), hearing impairment (HI), speech and language disorders, autism spectrum disorders (ASDs), and intellectual disability (ID). Furthermore, 6–9-year-old children were also assessed for attention deficit hyperactivity disorder (ADHD) and learning disorders (LDs). We standardized sample characteristics as per Census of India 2011 to arrive at district level and all-sites-pooled estimates. Site-specific prevalence of any of seven NDDs in 2–<6 year olds ranged from 2.9% (95% CI 1.6–5.5) to 18.7% (95% CI 14.7–23.6), and for any of nine NDDs in the 6–9-year-old children, from 6.5% (95% CI 4.6–9.1) to 18.5% (95% CI 15.3–22.3). Two or more NDDs were present in 0.4% (95% CI 0.1–1.7) to 4.3% (95% CI 2.2–8.2) in the younger age category and 0.7% (95% CI 0.2–2.0) to 5.3% (95% CI 3.3–8.2) in the older age category. All-site-pooled estimates for NDDs were 9.2% (95% CI 7.5–11.2) and 13.6% (95% CI 11.3–16.2) in children of 2–<6 and 6–9 year age categories, respectively, without significant difference according to gender, rural/urban residence, or religion; almost one-fifth of these children had more than one NDD. The pooled estimates for prevalence increased by up to three percentage points when these were adjusted for national rates of stunting or low birth weight (LBW). HI, ID, speech and language disorders, Epi, and LDs were the common NDDs across sites. Upon risk modelling, noninstitutional delivery, history of perinatal asphyxia, neonatal illness, postnatal neurological/brain infections, stunting, LBW/prematurity, and older age category (6–9 year) were significantly associated with NDDs. The study sample was underrepresentative of stunting and LBW and had a 15.6% refusal. These factors could be contributing to underestimation of the true NDD burden in our population.

**Conclusions:**

The study identifies NDDs in children aged 2–9 years as a significant public health burden for India. HI was higher than and ASD prevalence comparable to the published global literature. Most risk factors of NDDs were modifiable and amenable to public health interventions.

## Abstract 2

Narendra K Arora and colleagues estimate the burden of neurodevelopmental disorders in children aged 2-9 years in 5 regions of India, and identify prevalent risk factors.

## Author summary

**Why was this study done?:**

- Neurodevelopmental disorders (NDDs) compromise the development and attainment of full social and economic potential at individual, family, community, and country levels.
- Lack of robust evidence regarding burden and risk factors impedes policy and programmatic action for these conditions.
- Given the widespread prevalence of known risk factors, the anticipated burden of NDDs in children in India could be considerably high, but adequate information is not available.

**What did the researchers do and find?:**

- In this population based study, the prevalence of NDDs among 2–9-year-olds was estimated across five geographically diverse sites in India: North-Central (Palwal), North (Kangra), East (Dhenkanal), West (North Goa), and South (Hyderabad).
- We assessed 3,964 chidren (2–<6 years: 2,057; 6–9 years: 1,907) for seven common NDDs: vision impairment (VI), epilepsy (Epi), neuromotor impairments including cerebral palsy (NMI-CP), hearing impairment (HI), speech and language disorders, autism spectrum disorders (ASDs), and intellectual disability (ID). Two additional NDDs (attention deficit hyperactivity disorder [ADHD] and learning disorders [LDs]) were also assessed in 6–9-year-old children.
- Prevalence of NDDs varied between sites. Site-specific prevalence of any of seven NDDs in 2–<6year olds ranged between 2.9% and 18.7% and for any of nine NDDs in the 6–9-year-old children from 6.5% to 18.5%. About one-fifth of these children had two or more NDDs.
- HI and ID were the most common NDDs.
- The risk factors for childhood NDDs were as follows: children with history of delivery at home, delayed crying or difficult breathing at birth (perinatal asphyxia), neonatal illness requiring hospitalization, neurological/brain infections, low birth weight (LBW) (<2.5 kg) and/or birth before 37 weeks of gestation (prematurity), and stunting. NDDs were also likely to be more frequent in older children (6–9 year age category).

**What do these findings mean?:**

- Almost one in eight children of the age 2–9 years have at least one of the nine NDDs; this is a conservative estimate, and actual burden might be higher due to limitations of the study.
- The data suggested that the NDD burden can be substantially reduced in India by addressing the risk factors which are amenable to public health interventions.

## Introduction

“Neurodevelopment is a dynamic inter-relationship between genetic, brain, cognitive, emotional and behavioural processes across the developmental lifespan. Significant and persistent disruption to this dynamic process through environmental and genetic risk can lead to neurodevelopmental disorders and disability” [1]. Low-income communities and children living in poverty are disproportionately affected by NDDs [2]. Communities most vulnerable to NDDs often lack disease burden estimates to formulate policy decisions and implement programs to address NDDs [3]. To better understand the spectrum of childhood NDDs, there is a need to utilize valid and practical screening methodologies based on globally accepted disease definitions [4]. To date, global health policy makers have relied on national census disability data, even though such an approach grossly underestimates disability prevalence in children [5]. Censuses usually restrict themselves to the identification of gross and visible disabilities only and utilize nonspecialized assessors and diagnostic tools [5,6]. Global and societal leaders have urged that nations promote awareness of children with disabilities and advocate for their healthcare services [3]. The United Nations General Assembly [7] and Agenda for Sustainable Development [8] consider childhood disability an integral part of the global development agenda and promote the use of evidences that address national and regional contexts and are disaggregated by gender and age.

India has the world’s largest birth cohort (about 26 million) and is experiencing dynamic improvements in both infant and child survival [9,10]. The neonatal, infant and under-five mortality rates in India have shown significant decline during the last decade; improved survival of children and infants with high risk for NDDs is likely to result in higher community prevalence, lest interventions are instituted concomitantly [11–13]. To address the challenge of inadequate data, we convened a series of working groups to design and conduct a study of population-level prevalence of childhood NDDs in India through a transdisciplinary approach. Our aim was to determine the prevalence of NDDs among children aged 2–9 years in India and identify potential demographic and individual risk factors that could be applied at the national level. We decided to assess these children for nine common NDDs: vision impairment (VI), epilepsy (Epi), neuromotor impairments including cerebral palsy (NMI-CP), hearing impairment (HI), speech and language disorders, autism spectrum disorders (ASDs), intellectual disability (ID), attention deficit hyperactivity disorder (ADHD), and learning disorders (LDs).

## Methods

We have reported this study as per the Strengthening the Reporting of Observational Studies in Epidemiology (STROBE) guidelines (S1 STROBE Checklist).

### Study design

We conducted a cross-sectional survey at five sites in India.Within each site, we used probability proportionate to population size (PPS) cluster sampling to select households for the survey.

### Investigator group

A Technical Advisory Group (TAG) comprising 55 transdisciplinary experts (51 from India and four from the United States of America) provided guidance and oversight throughout the project. The TAG included experts in paediatrics (paediatric neurology, developmental paediatrics, general paediatrics), epidemiology, public health, social science, biostatistics, child psychology, oto-rhino-laryngology, ophthalmology, and child psychiatry from 18 institutions. The central coordinating office for the study was located at the INCLEN Executive Office in New Delhi.

### Settings

Considering the major geographic (North, South, East, West), demographic, sociocultural and topographic (hilly terrain, plains, coastal regions) population heterogeneity in India, we selected one district each from five states across the country with the following characteristics: Kangra in Himachal Pradesh (Northern India; Himalayan terrain; altitude ranges from 427 to 6,401 metres; population: 94.3% rural; literacy: 85.7%); Palwal in Haryana (North-Central India; plains; population: 77.3% rural; literacy: 69.3%); Dhenkanal in Odisha (Eastern India; plains; population: 14.0% tribal, 90.0% rural; literacy: 70.6%); Hyderabad in erstwhile Andhra Pradesh (now Telangana) (Southern India; plains; population: 100.0% urban; literacy: 83.3%); and North Goa (West India; coastal; population: 59.9% urban; literacy: 90.7%) (demographic details taken from Census of India, 2011) [14]. The technical capacity of the potential collaborators and availability of infrastructural facilities and logistical support also contributed to decisions regarding site selection. Villages and municipal wards as listed in the Census register (Census of India, 2011) were the primary sampling units to select clusters of rural and urban areas, respectively. Fifty clusters were identified at three sites. At Hyderabad and North Goa sites, only 25 clusters were drawn due to limited finance availability. In total (five sites pooled), we surveyed 200 clusters (42 urban and 158 rural). The data was collected between 5 December 2011 and 27 September 2012.

### Participants and recruitment

An “eligible participant” was defined as a child in the age range of 2–9 years (24–119 months) in the household. We requested the parent(s)/legal guardian(s) to provide written informed consent for participation in the study and to visit the study clinic for the diagnostic work-up of the child. We recruited 20 participants—five boys and five girls each from the age categories 2–<6 years and 6–9 years—in every cluster. A landmark located centrally in the cluster (e.g., temple, mosque, church, market place, panchayat office, central place) was identified with the help of local people. The clusters were virtually divided into two halves, for enrolling boys from one half and girls from the other. The direction for enumeration and choice of the first household of each half of the cluster was decided through a random number. For households with more than one eligible child, we recruited the eldest child in the even-numbered clusters and the youngest child in odd-numbered clusters. One of the parents of the child was the preferred respondent. Children who dropped out from diagnostic assessment after initial enrolment were replaced from the same cluster (matched for age category and gender) by continuing enumeration of consecutive households.

Two research teams were engaged for data collection at each site: (a) the field team comprising one physician and two social scientists, and (b) the diagnostic team comprising one physician, one audiologist/speech therapist, and two psychologists. The field teams identified the eligible participants, obtained consent, collected information on demographic characteristics including socioeconomic variables and risk factor variables at the participants’ residence, and thereafter mobilized them to the partner institution (tertiary care hospital) study clinic for detailed NDD work-up. At the hospital, under the supervision of the site investigators, members of the diagnostic team conducted the following: (i) physician assessments for VI, Epi, and NMI-CP; (ii) audiologist/speech therapist assessment for HI and speech and language disorders; and (iii) psychologist assessment for ASD, ID, ADHD, and LD.

### Training of the research teams

The TAG prepared standard operating procedures and training modules for the research teams. Three-day structured training programs were conducted by a team of four multidisciplinary TAG members at partner hospitals for both field and diagnostic teams; 50% training time was allocated for hands-on activities in the field. Thereafter, one quality assurance visit per site by the TAG members was organized (all within eight weeks of starting the field work) to ensure adherence to the assessment protocols. Additionally, the central coordinating site conducted teleconferences with the site teams each time they completed data collection from two clusters.

### Study variables and measurement

Personal details and information on sociodemographic variables, asset variables for Standard of Living Index (SLI), and biological and nutritional “risk factor” variables relevant for NDDs were obtained for study participants. At the study clinics, every participant was assessed for NDDs as per the Diagnostic and Statistical Manual of Mental Disorders, Fourth Edition, Text Revision (DSM-IV-TR) guidelines. Prior validation, reported use among Indian participants, and feasibility of application in community settings were the criteria used by the TAG for zeroing in on the instruments used in the study (Table 1). As diagnostic tools for Epi, NMI-CP, ASD, and ADHD satisfying the above criteria were not available, the INCLEN investigator team developed culturally adapted and context-relevant diagnostic tools for these four conditions and validated them against globally established guidelines and tools [15–18]. The instruments and examination formats for the NDDs were compiled into a child assessment booklet (CAB) and applied consistently on all participants across the study sites. NDD evaluations followed a specific algorithm to determine the clinical diagnosis. Since ADHD and LD could be assessed only in older children (6–9-year-olds), the younger study participants (2–<6-year-olds) were assessed for seven NDDs, and children aged 6–9 years were assessed for all nine NDDs. A subcommittee of the TAG reviewed the data for resolution of diagnostic ambiguity.

**Table 1:** Diagnostic assessments for NDDs in study participants.

NDD assessed	Diagnostic instrument	Necessary precondition	Method
**Physician’s Station (Time taken: about 1 hour)**			
**VI**	Cardiff Visual Acuity Test (for <3-year-old children) [19,20]; LogMar E Chart (for ≥3-year-old children) [21,22]	Visual acuity <6/18 in the better eye (uncorrected) was diagnosed as VI	Test/examination
**Epi**	INDT–EPI [15]	**The following conditions were not considered as “epilepsy”**: febrile seizures, seizures occurring within seven days of a head injury or cerebrovascular hemorrhage, seizures during the course of an active central nervous system infection, and in metabolic disturbances like hypoglycemia, hypocalcaemia, hyponatremia, and hypoxia	Interview
**NMI–CP**	INDT–NMI [16]		Interview, observation and examination
**Audiologist/Speech therapist’s Station (Time taken: about 1 hour)**			
**HI**	OAE Audiometry [23]	**Exclusions:** Impacted cerumen, foreign body, visible external ear disease, and ear discharge (otitis media); Permanent unaided hearing threshold of ≥35 dB nHL in the better ear was diagnosed as HI	Test /examination
**Speech and Language Disorders**	LPT [24]	Age ≥3 years; **Exclusions:** HI, ID, and ASD	Test and observation
**Psychologists’ Station (Time taken: about 3 hours)**			
**ASDs**	INDT–ASD [17]		Interview and observation
**ID**	VSMS [25] for calculating SQ; Stanford Binet Intelligence Scale (Kulshrestha–Indian adaptation) [26] for calculating IQ	Children with SQ and IQ ≤70 were diagnosed as intellectually disabled	Interview and observation
**ADHD**	INDT–ADHD [18]	Age ≥6 years; **Exclusions:** ID and ASD	Interview and observation
**LDs**	GLAD [27]	Age ≥6 years; IQ ≥85; Presently going to school or had attended school for at least 6 months (in case of school drop–outs) or studying at home using conventional teaching methods; **Exclusions:** ID, HI and VI	Test and observation

**Abbreviations:** ADHD, attention deficit hyperactivity disorder; ASD, autism spectrum disorder; Epi, epilepsy; GLAD, Grade Level Assessment Device; HI, hearing impairment; ID, intellectual disability; INDT–ASD, INCLEN Diagnostic Tool for Autism Spectrum Disorders; INDT–EPI, INCLEN Diagnostic Tool for Epilepsy; INDT–NMI, INCLEN Diagnostic Tool for Neuro–Motor Impairments; IQ, intelligence quotient; LD, learning disorder; LPT, linguistic profile test; NDD, neurodevelopmental disorder; NMI-CP, neuromotor impairments including cerebral palsy; OAE, oto-acoustic emission; SQ, social quotient; VI, vision impairment; VSMS, Vineland Social Maturity Scale.

### Sample size

Based on previous NDD prevalence reports from South Asian countries [28], we assumed that about 10% of children aged 2–9 years could have at least one NDD. For an admissible absolute error of ±2% at 95% confidence level, we arrived at the sample size of 864 participants per site. Taking into account potential nonresponses and operational feasibility issues, 1,000 children were enrolled per site. Due to paucity of resources and personnel, we reduced the sample size to half at two sites (Hyderabad and North Goa); this increased the admissible absolute error to ±2.65% for these sites.

### Analysis

For analysis, we used Microsoft Excel 2010 and STATA v12.0 [29]. Our analysis was guided by a prospectively written statistical analysis plan at the time of preparation of this proposal for funding (S1 Text). Nutritional status of participants (somatic measurements) was adjusted for WHO Z-scores using WHO AnthroPlus software [30].We computed the SLI score for every participant household, assigning scores for assets and household characteristics as per National Family Health Survey-3 (NFHS-3) 2005–2006 (Demographic and Health Survey for India–3^rd^ Round) [31] and, using NFHS-3 state-specific cutoffs, assigned quintile status to each household. Thus, we generated both SLI scores and quintile statuses for the participants. The study population was stratified according to age category (2–<6-year-old and 6-9-year-old), gender (boy/girl), place of residence (rural/urban), and religion (Hindu/non-Hindu) and standardized for these variables as per Census of India 2011 to arrive at respective district level estimates. The data from the five sites was pooled and weighed against Census 2011 population figures aggregated from respective districts for age, gender, place of residence (rural/urban), and religion (Hindu/non-Hindu). As Census counts were not available according to nutritional status of individuals, standardizing for stunting and low birth weight (LBW) could not be done.

The complex survey module of STATA (SVYSET and SVY) was used for estimating the prevalence and risk factors.

#### Risk factor analysis

To identify risk factors associated with presence of NDDs, we performed multivariable logistic regression modelling on the background of SVY command of STATA. The independent variables for the regression were adjusted into the model using subject knowledge. These were history of consanguinity, mental or neurological illness in the family, medical complications during pregnancy, chorioamnionitis in the mother at the time of index pregnancy, birth order (≥3 versus <3), multiple pregnancies, place of delivery (institutional versus noninstitutional delivery), history of perinatal asphyxia, serious neonatal illness (requiring hospitalization), traumatic brain injury, postnatal neurological infections (e.g., meningitis, encephalitis), LBW and/or prematurity, and stunting. Gender (boy/girl), place of residence (rural/urban), education status of the respondent (“never been to school”/“ever been to school”), religion (Hindu/non-Hindu), caste (SC-ST/rest), SLI score (continuous variable), and age category (6–9-year-old versus 2–<6-year-old) were also adjusted into the model. We estimated the population attributable fraction (PAF) [32,33] for the final multivariable logistic model.

### Data cleaning and processing

All CABs were scrutinized at two levels: by the research teams and the site investigators for consistency and completeness of diagnostic assignment and again at the central coordinating office. Discrepancies discovered at central coordinating office were resolved through communication with the site investigators (weekly teleconference) and TAG review (quality assurance site visits and reviewing of CAB by TAG subcommittee). TAG members rechecked the diagnostic assignment in 10% of CABs of participants (chosen randomly) with NDD and an equal number without NDD and did complete review of all participants labelled as “indeterminate.”

### Consent

Informed written consent was solicited from parents/guardian of the children prior to inclusion in the study.

### Ethics considerations

Ethics approval was obtained from the institutional review boards of India-CLEN (Indian Network of INCLEN) and the participating sites. The study was conducted in compliance with the World Medical Association (WMA) Declaration of Helsinki [34]. Children identified with “any NDD” and/ or other medical conditions during the course of the study were referred to the nearest appropriate tertiary care centre for management and followed up with due counselling.

## Results

Table 2 illustrates the study recruitment profile for each site. Overall, the field teams approached families of 4,739 children, of which 739 (15.6%) refused to participate. After enrolling the targeted 4,000 children, 181 (4.5%) refused to complete diagnostic assessment, of which 158 (4.0%) could be replaced with age category and gender-matched children from the same cluster. Post hoc analyses revealed that dropouts and their replacements also matched for anthropometric measurements and sociodemographic characteristics (S1 Table). The NDD assessment could be done in 3,977 children (83.9% of the total approached; 99.4% of those enrolled).

**Table 2:** Study recruitment profile.

	Palwal	Kangra	Dhenkanal	Hyderabad	North Goa	All sites pooled
**Sampling frame**	**207,979**	**193,910**	**166,234**	**507,099**	**90,700**	**1,165,922**
**Number of clusters**	50	50	50	25	25	200
**Number of children identified as eligible for recruitment**	1,189	1,181	1,185	597	587	4,739
**Refused to participate**	189 (15.9%)	181 (15.3%)	185 (15.6%)	97 (16.2%)	87 (14.6%)	739 (15.6%)
**“No time to participate”**	26 (13.8%)	24 (13.3%)	44 (23.8%)	6 (6.2%)	36 (4.1%)	136 (18.4%)
**“Will not travel to study clinic”**	46 (24.3%)	41 (22.7%)	48 (25.9%)	42 (43.3%)	51 (5.9%)	228 (30.9%)
**“Primary care provider not at home”**	90 (47.6%)	92 (50.8%)	45 (24.3%)	13 (13.4%)	0 (0.0%)	240 (32.3%)
**No specific reason**	27 (14.3%)	24 (13.3%)	48 (25.9%)	36 (37.1%)	0 (0.0%)	135 (18.3%)
**Consented and Enrolled**	**1,000**	**1,000**	**1,000**	**500**	**500**	**4,000**
Refused to complete diagnostic assessment after enrolment*	46 (4.6%)	37 (3.7%)	64 (6.4%)	34 (6.8%)	0 (0.0%)	181 (4.5%)
**Replaced (age category and gender matched) for those who did not come for assessment**	44 (4.4%)	37 (3.7%)	45 (4.5%)	32 (6.4%)	0 (0.0%)	158 (4.0%)
**Available for diagnostic assessment**	998 (99.8%)	1,000 (100.0%)	981 (98.1%)	498 (99.6%)	500 (100.0%)	3,977 (99.4%)
**Assessment incomplete/Not properly done (dropped from analysis)**	0 (0.0%)	3 (0.3%)	0 (0.0%)	3 (0.6%)	7 (1.4%)	13 (0.3%)
**Available for analysis**	**998** **(99.8%)**	**997** **(99.7%)**	**981** **(98.1%)**	**495** **(99.0%)**	**493** **(98.6%)**	**3,964** **(99.1%)**

* Reasons for noncompletion after enrollment (*N* = 181): “no time,” 133 (73.5%); “will not travel to study clinic,” 48 (26.5%). Values within parentheses indicate percentages.

During data review at the central coordinating office, errors in score computation done at field sites were corrected in 358 out of 3,977 records (9%); 179 (4.5%) CABs were returned to the primary site for completion, and all were obtained back. For quality assurance, the TAG reviewed 50 random CABs per site for cases labelled with or without NDD, respectively, and found no disagreements. The TAG reviewed an additional 174 CABs labelled as “indeterminate”; of these, a definitive diagnosis was made in 161, and the remaining 13 were excluded (due to incomplete information) from the study analysis, leaving a final number of 3,964 participants (2,006 boys and 1,958 girls) for analysis.The background characteristics of study participants are summarized in Table 3.

**Table 3:** Background characteristics of study participants.

Characteristics	Study sites (Percentage of participants)					
	Palwal (*N* = 998)	Kangra (*N* = 997)	Dhenkanal (*N* = 981)	Hyderabad (*N* = 495)	North Goa (*N* = 493)	All sites pooled (*N* = 3,964)
**Place of residence: Rural**	100.0	91.6	90.0	0.0	68.0	79.0
**Gender: Female**						
**2–<6-year-olds**	49.4	49.1	49.9	46.3	50.2	49.2
**6–9-year-olds**	49.2	49.7	50.4	48.7	50.0	49.7
**Family type: Nuclear**	65.8	71.6	78.3	91.7	63.9	73.3
**Religion: Non–Hindu**	16.4	3.7	1.2	25.7	11.4	10.1
**Ethnicity: SC-ST**	25.3	25.3	38.0	27.4	18.5	27.8
Head of household: Literate*	78.0	88.6	93.4	75.2	89.7	85.5
**Either parent: Employed**	99.0	88.0	99.7	91.2	86.0	93.9
**Either parent as the respondent**	55.4	91.5	99.1	81.4	97.2	83.7
Standard of Living Index**						
**Poor**	20.0	5.0	15.8	64.8	7.4	14.2
**Lower Middle Class**	26.7	23.0	12.4	25.6	19.1	13.0
**Middle Class**	25.3	41.0	10.2	9.0	33.7	21.0
**Upper Middle Class**	18.5	24.6	38.1	0.4	26.0	33.0
**Rich**	9.6	7.0	23.5	0.4	14.0	19.0

*Can write his/her name in any language.

**Computed against quintile distribution of respective state as per SLI.

**Abbreviations:** SC-ST, scheduled caste/tribe.

### Prevalence of NDDs

Overall, we found 475 of 3,964 children (between the ages of 2 and 9 years) had at least one NDD (12.0% [95% CI 11.0%–13.0%]). Among children with NDDs, 21.7% (95% CI 18.1%–25.7%) had two or more NDDs; children with ASD (79.6%), NMI-CP (74.2%), ID (56.9%), and Epi (55.1%) most frequently had coexisting NDD(s) (S2 Table). The district level prevalence estimates for NDDs across the five sites, upon weighing for age category, gender, place of residence (rural/urban), and religion (Hindu/non-Hindu) as per Census 2011, is provided in Table 4. There was site-specific variation in the prevalence: Dhenkanal had the lowest and Palwal the highest prevalence of NDDs. HI, ID, speech and language disorders, Epi, and LDs were among the most common NDDs across the sites.

**Table 4:** Prevalence estimates of NDDs for the five study districts according to age categories*.

Diagnosis↓ Age category (in years)	Sites											
	Palwal		Kangra		Hyderabad		Dhenkanal		North Goa		All sites pooled	
	2–<6	6–9	2–<6	6–9	2–<6	6–9	2–<6	6–9	2–<6	6–9	2–<6	6–9
Any NDD** (at least 1)	18.7	18.5	9.5	15.3	9.6	14.4	2.9	6.5	8.0	16.0	9.2	13.6
	(14.7–23.6)	(15.3–22.3)	(7.0–12.8)	(11.8–19.7)	(6.2–14.6)	(9.9–20.4)	(1.6–5.5)	(4.6–9.1)	(4.7–13.2)	(11.2–22.4)	(7.5–11.2)	(11.3–16.2)
**>1 NDD**	4.3	5.3	1.6	3.3	3.3	2.4	0.4	0.7	1.5	3.0	2.3	2.6
	(2.2–8.2)	(3.3–8.2)	(0.8–3.1)	(2.0–5.4)	(1.7–6.4)	(0.8–7.1)	(0.1–1.7)	(0.2–2.0)	(0.5–4.1)	(1.4–6.2)	(1.5–3.4)	(1.7–4.0)
**VI**	0.3	0.0	1.0	0.5	1.0	0.9	0.9	0.7	0.0	0.8	0.7	0.6
	(0.0–1.3)	(0.0–0.7)	(0.4–2.4)	(0.1–1.8)	(0.2–4.9)	(0.1–6.2)	(0.3–2.8)	(0.2–2.0)	(0–1.5)	(0.2–3.6)	(0.3–1.8)	(0.2–1.9)
**Epi**	1.4	3.6	0.8	2.6	1.6	2.7	0.6	0.9	0.2	1.9	1.1	2.2
	(0.7–2.9)	(2.1–6.2)	(0.4–2.0)	(1.4–4.9)	(0.7–3.7)	(1.0–7.0)	(0.2–1.9)	(0.3–2.3)	(0.0–1.8)	(0.8–4.7)	(0.6–1.7)	(1.4–3.6)
**NMI-CP**	3.8	2.0	1.8	1.9	2.9	1.5	0.4	0.2	1.6	1.0	2.1	1.3
	(1.8–7.8)	(0.8–4.7)	(0.8–3.7)	(0.9–4.3)	(1.3–6.4)	(0.4–5.7)	(0.1–1.7)	(0.0–1.5)	(0.4–7.1)	(0.3–3.7)	(1.3–3.4)	(0.7–2.4)
**HI**	9.9	7.9	2.2	2.6	3.4	2.7	0.6	1.1	0.2	0.2	3.3	2.6
	(7.2–13.6)	(5.5–11.2)	(1.1–4.7)	(1.3–5.2)	(1.4–7.7)	(1.3–5.4)	(0.1–2.7)	(0.4–3.0)	(0.0–1.8)	(0.0–1.8)	(2.3–4.8)	(1.9–3.7)
**Speech and language disorders**	2.0	1.6	2.9	2.6	0.5	0.3	0.0	0.2	5.9	4.3	1.6	1.6
	(1.0–3.8)	(0.8–3.2)	(1.5–5.3)	(1.2–5.3)	(0.0–3.6)	(0.0–2.1)	(0.0–0.7)	(0.0–1.4)	(3.2–10.6)	(2.3–8.0)	(1.0–2.4)	(1.0–2.7)
**ASDs**	1.7	1.9	0.6	0.9	1.3	2.1	0.6	1.0	0.5	0.2	1.0	1.4
	(0.6–4.8)	(0.8–4.6)	(0.2–1.7)	(0.3–3.0)	(0.5–3.3)	(0.5–8.2)	(0.2–1.9)	(0.3–3.7)	(0.1–2.0)	(0.0–1.8)	(0.6–1.6)	(0.6–3.2)
**ID**	5.6	6.6	2.8	5.7	4.7	8.0	0.6	0.7	1.5	1.2	3.1	5.2
	(3.3–9.4)	(4.7–9.3)	(1.6–5.1)	(3.6–8.4)	(2.6–8.2)	(4.6–13.7)	(0.2–2.7)	(0.2–2.0)	(0.5–4.1)	(0.4–3.7)	(2.2–4.2)	(3.4–7.7)
ADHD**	-	0.8	-	2.3	-	0.5	-	0.7	-	1.9	-	1.0
		(0.3–2.0)		(1.1–4.7)		(0.1–2.2)		(0.2–2.0)		(0.8–4.5)		(0.6–1.5)
LDs**	-	1.5	-	1.2	-	-	-	2.0	-	7.6	-	1.6
		(0.5–4.5)		(0.4–3.3)				(1.0–3.9)		(3.9–14.2)		(1.0–2.5)

*Weighed according to district population for age category, gender, place of residence (rural/urban), and religion (Hindu/non-Hindu) as per Census of India, 2011.Values in parentheses indicate 95% CI.

**ADHD and LD were assessed only in the 6–9 year age category, and therefore, prevalence for 2–<6 and 6–9 year age category were calculated separately.

**Abbreviations:** ADHD, attention deficit hyperactivity disorder; ASD, autism spectrum disorder; CI, confidence interval; Epi, epilepsy; HI, hearing impairment; ID, intellectual disability; LD, learning disorder; NDD, neurodevelopmental disorder; NMI-CP, neuromotor impairments including cerebral palsy; VI, vision impairment.

There were no significant differences in the all-sites-pooled data for gender, place of residence, and religion weighed as per Census 2011: prevalence among boys 12.4% (95% CI 10.2%–15.0%) versus 10.2% (95% CI 8.4%–12.2%) in girls (*p* = 0.146); rural areas 12.6% (95% CI 11.4%–13.9%) versus 10.1% (95% CI 7.9%–12.8%) from urban areas (*p* = 0.085); and in non-Hindu families 11.7% (95% CI 8.5%–15.9%) versus those from Hindu families 11.0% (95% CI 9.7%–12.5%) (*p* = 0.727).

#### Clinical profile of NDDs

Of the 62 cases of NMI-CP, 46.8% had spastic cerebral palsy (CP), 22.6% had neuromuscular disorder (NMD), and 30.6% had other NMIs. ID (*N* = 144) was assessed as mild (27%), moderate (13.8%), or severe and profound (3.5%); in the remaining 55.6% (80/144), severity could not be determined, primarily due to associated comorbidities (62/80; 77.5%). Out of 44 cases of ASD, 52.3% were diagnosed as autism, 2.3% as Asperger, 38.0% as pervasive developmental disorder not otherwise specified (PDD-NOS), and 7.1% were diagnosed to have childhood disintegrative disorders (CDDs). In 27 cases of ADHD, 44.4% had inattention, 11.1% had hyperactivity/impulsivity, and the remainder (44.4%) were of mixed type. The assessment tool for Epi was not designed to determine the type of Epi.

### Risk factors analysis

S3 Table enlists risk factor information for the study participants with and without NDDs. Table 5 provides the independent risk factors in the multivariable regression model: noninstitutional delivery, history of perinatal asphyxia, history of neonatal illness, postnatal neurological (brain) infections, stunting, LBW (<2.5 kg)/prematurity (gestation <37 weeks), and older (6–9 years) age category were statistically significantly associated with “any NDD.” The PAF was 36.8% (95% CI 27.2%–45.1%) for modifiable and statistically significant risk factors. We also undertook multivariable analysis for identifying risk factors for specific NDDs (S4 Table). The risk factors for “any NDD” were variably present with specific NDDs as well.

**Table 5:** Multivariable logistic regression analysis for risk factors for NDDs^#^.

Risk factors	Reference	AOR 95% CI	*p* value	PAF 95% CI**
**Modifiable risk factors**				**43.8 (32.9**–**52.9)**
** Consanguinity**	No consanguinity	1.4 (0.7–2.8)	0.343	2.2 (0.03–4.3)
** Neurological or mental illness in the family**	No neurological or mental illness in the family	1.0 (0.7–1.6)	0.823	3.4 (0.2–6.4)
** Medical complications during pregnancy**	No medical complications during pregnancy	1.0 (0.6–1.9)	0.938	2.4 (0.0–5.6)
** Chorioamnionitis**	No chorioamnionitis	1.5 (0.8–3.0)	0.235	2.6 (0.5–4.7)
** Birth order ≥3**	Birth order <3	1.4 (0.9–2.2)	0.129	11.3 (6.6–15.8)
** Multiple pregnancies**	Single pregnancy	0.6 (0.3–1.4)	0.282	0.05 (0.0–1.8)
** Place of delivery (Non-institutional)**	Institutional	1.5 (1.0–2.3)	0.042*	18.2 (11.6–24.4)
** Perinatal asphyxia**	No perinatal asphyxia	1.9 (1.0–3.5)	0.039*	6.3 (3.3–9.2)
** Neonatal illness (requiring hospitalization)**	No neonatal illness	2.2 (1.5–3.1)	<0.001*	10.4 (6.9–13.8)
** Traumatic brain injury**	No traumatic brain injury	1.8 (0.9–3.4)	0.087	3.5 (1.4–5.5)
** Post-natal neurological/brain infections**	No Post-natalneurological/ brain infection	3.3 (1.3–8.8)	0.011*	3.4 (1.9–4.9)
** Stunting**	No stunting	1.6 (1.1–2.4)	0.012*	11.8 (6.4–17.0)
** LBW (<2.5kg)/prematurity (gestation <37weeks)**	Birth weight ≥2.5 kg and gestation ≥37 weeks	1.6 (1.1–2.5)	0.022*	7.2 (3.4–10.9)
**Nonmodifiable risk factors**				**31.5 (8.0**–**49.1)**
**Gender (Boy)**	Girl	1.2 (0.9–1.6)	0.335	13.1 (4.2–21.1)
**Place of Residence (Rural)**	Urban	0.7 (0.4–1.1)	0.090	18.7 (1.8–32.6)
**Education (never been to school)**	Ever been to school	1.0 (0.6–1.6)	0.983	11.2 (5.8–16.4)
**Religion (non-Hindu)**	Hindu	0.8 (0.5–1.3)	0.332	1.6 (0.0–4.6)
**Caste (SC-ST)**	Rest	1.2 (0.8–1.7)	0.345	0.9 (0.0–6.2)
**SLI score**	(Continuous variable)	0.99 (0.97–1.00)	0.082	
**Age category(6–9 years)**	2–<6 years	1.4 (1.0–1.9)	0.023*	14.8 (6.3–22.5)

^#^ Weighed according to national population for age category, gender, place of residence (rural/urban), and religion (Hindu/non-Hindu) as per Census of India, 2011.

* Statistically significant (*p* < 0.05).

** PAF for all modifiable and nonmodifiable factors taken together:76.9% (95% CI 45.6%–90.2%); PAF for statistically significant modifiable factors only: 36.8% (95% CI 27.2%–45.1%).

**Abbreviations:** AOR, adjusted odds ratio; LBW, low birth weight; NDD, neurodevelopmental disorder; SC-ST, scheduled caste/tribe; SLI, Standard of Living Index.

## Discussion

The study reports the prevalence of NDDs in children aged 2 to 9 years obtained through a population-based, multisite survey across five regions in India. The prevalence of NDDs varied across the five study sites despite using the same diagnostic tools with application of consistent methodology and training for the assessors. NDD prevalence might truly vary across regions, particularly in a large country like India, due to heterogenous distribution of risk factors and biological factors, if any. Dhenkanal, situated in central Odisha, with a sizeable tribal population, has high under-five mortality rate (80 per 1,000 live births) [35] and is endemic for hemoglobinopathies [36] and cerebral malaria (cerebral malaria in Odisha has up to six times higher risk of mortality among children) [37,38]. It has been reported that the risk of mortality in children with NDDs may be high in environments of poor economic development, weak health systems, and high child mortality [4,39]. The higher prevalence of HI in Palwal contributed to a higher overall prevalence of NDD in this site over others. According to an earlier study, repeated respiratory infections and high rates of chronic suppurative otitis media were associated with a high rate of deafness in 5–15-year-old children in the area [40].

Literature suggests that the prevalence of NDDs and their profiles vary considerably within and between geographies [4,41–45]; this has commonly been attributed to methodological variability [4] but can also be seen in studies that used common diagnostic tools and criteria and common data collection methods across sites. For example, based on parent-reported diagnosis, Boyle and colleagues [46] reported the prevalence of ASD in the United States of America to be 0.7%, but when systematic community-based assessment was done at 11 sites by the Autism and Developmental Disabilities Monitoring Network, overall prevalence of ASD was almost twice that of the previous study (1.5%) [47]. The Ten Questions (TQ) screen for disability was uniformly applied in a study of 18 low- and middle-income countries (LMICs); the prevalence of children aged 2–9 years at high risk for disability ranged between 3% (Uzbekistan) and 48% (Central African Republic), with a median prevalence of 23% [48]. In the two-phased studies with TQ in the similar age group, the prevalence of moderate to severe forms of disability varied between 0.5% and 9.4% in several LMICs [49–51].

In the Indian census form, there are four questions for collecting information on eight severe and obviously visible disabilities, including NDDs. According to Census of India 2011 data, the prevalence of all disabilities in the age category 0–4 years was 1.1%, and in the age category 5–9 years, it was 1.5% [52]. These figures were almost 10 times less than what we reported in the present study after a systematic, comprehensive, community-based assessment exclusively for NDDs; the study findings were likely to present a more factual picture of the population-based prevalence of NDDs in different parts of India.

The cumulative prevalence of NDDs was higher in the older age category (6–9 years) as compared to the younger age category (2–<6 years). According to published literature, observation of higher prevalence of NDDs with increasing age in childhood might be due to the following: (a) accumulation of childhood-onset causes and exposures, such as infections, injuries, and nutritional deficiencies; and (b) increased ability to recognize and diagnose conditions such as CP, ID, and behavioral disorders as age advanced [53]. In the regression model in which we fitted age as a categorical variable (6–9-year-old versus 2–<6-year-old, as a reference), it emerged as a statistically significant predictor of NDD (Table 5). We could have also possibly arrived at this observation because participants in the older age category were assessed for two additional NDDs (i.e., ADHD and LD).

Almost one-fifth of the children with NDDs had another comorbid NDD. Similarly, there was clustering of risk factors in the same individuals. We looked for the various modifiable risk factors significantly associated with occurrence of any or a cluster of NDDs in the same individual. The odds of any NDD among children with two or more risk factors and three or more risk factors were 2.1 (95% CI 1.5–2.9) and 2.6 (95% CI 1.7–4.0), respectively. Walker and colleagues have suggested that cumulative exposure to risks starting from the prenatal period affects the developmental trajectories and lays the foundation for NDDs; these risk factors therefore require early identification and integrated interventions [54]. The frequent coexistence of ASD, NMI-CP, ID and/or Epi in (S2 Table) could be suggestive of common precursor events [55].

The results of the study have to be interpreted in the light of its limitations; 15.6% refusal to participate might be reflective of sociobehaviroal complexities associated with a child with NDD in the family (e.g., disability, fear, denial, guilt, blame, different dimensions of stigma and discrimination) that could in turn influence participation in such diagnostic evaluations [56,57]. The study sample was not representative of India (S5 Table); particularly, the sample was underrepresentative of stunting and LBW, and that could be contributing to underestimation of the true NDD burden in the study population. The stunting and LBW status of the children were not available in the Census data and therefore could not be adjusted together with other variables like age, gender, place of residence, and religion. The national rates (NFHS-4, 2015–2016) of stunting (38.4%) and LBW (18.2%) were available, and when these were applied separately to all-site-pooled estimates, prevalence of NDD in the 2–<6 year age category increased from 9.2% to 10.9% and 12.3%, respectively. Similarly, in the 6–9 year age group, weighing for these conditions led to increase in the estimates from 13.6% to 16.8% and 14.0%, respectively. The study was not designed to determine the social bias of “gender-selective treatments” on child survival, growth, and development [58]. The group has now validated a revised version of the Epi instrument to ascertain the types of Epi, including some of the ones not detected with the present tool [59].

Our study provided the first population-based multisite data on the burden of NDDs in 2–9-year-old children from India. We developed four new diagnostic tools that were culturally adapted and validated against international guidelines that use global normative data [15–18]; the tools were applied in the field by trained professionals with stringent quality control frameworks. While measurement of clinical conditions such as lack of vision or Epi is fairly comparable to other international measures (and prevalence rates are thus directly comparable), the same does not apply to the assessment of cognitive, attention, and learning skills, in which disability estimates would likely be substantially different if high-income population norms would be applied instead of an Indian reference population.

The study highlighted NDDs in children as an important public health challenge of considerable significance with substantial within-country variations. Most of the significant risk factors identified through the study were modifiable and could potentially be addressed by investments in public health to improve maternal/newborn care and child nutrition. Several emerging economies have been able to address these risk factors effectively [60]. The findings were important in the context of the Universal Health Care (UHC) agenda of the Government of India and the recently launched national child health program (“Rashtriya Bal Swasthya Karyakram”) [61]. These data will have relevance for India’s response to the needs of the children who are most vulnerable and their families and might also inform the policies of other countries that share similar socioeconomic milieu and healthcare situations, particularly in South Asia [54,62].

## Supporting information

S1 STROBE Checklist (DOCX)

S1 Table Comparison of “Drop-out” (*N* = 181) and “Replacement” (*N* = 158) participants in the study sites. (DOCX)

S2 Table Coexisting NDDs (by percentage) in study participants with at least one NDD. NDD, neurodevelopmental disorder.
(DOCX)

S3 Table Distribution of the risk factors among children with and without NDDs. NDD, neurodevelopmental disorder.
(DOCX)

S4 Table Multivariable logistic regression analysis for risk factors for specific NDDs in children aged 2–9 years. NDD, neurodevelopmental disorder.
(DOCX)

S5 Table Comparison of demographic features of the participant population with national databases. (DOCX)

S6 Table Summary description of four diagnostic tools developed and validated by INCLEN team for the study. (DOCX)

S1 Text Prospectively written statistical analysis plan. (DOCX)

S1 Data Palwal. (DTA)

S2 Data Kangra. (DTA)

S3 Data Dhenkanal. (DTA)

S4 Data Hyderabad. (DTA)

S5 Data Goa. (DTA)

S6 Data All. (DTA)

S7 Data Analysis file. (DO)

## Abbreviations

ADHD: attention deficit hyperactivity disorder
AOR: adjusted odds ratio
ASD: autism spectrum disorder
CAB: child assessment booklet
CDD: childhood disintegrative disorder
CI: confidence interval
CP: celebral palsy
DSM-IV-TR: Diagnostic and Statistical Manual of Mental Disorders, Fourth Edition, Text Revision
Epi: epilepsy
GLAD: Grade Level Assessment Device
HI: hearing impairment
ID: intellectual disability
INDT–ASD: INCLEN Diagnostic Tool for Autism Spectrum Disorders
INDT–EPI: INCLEN Diagnostic Tool for Epilepsy
INDT–NMI: INCLEN Diagnostic Tool for Neuro–Motor Impairments
IQ: intelligence quotient
LBW: low birth weight
LD: learning disorder
LMIC: low- and middle-income country
LPT: linguistic profile test
NDD: neurodevelopmental disorder
NFHS-3: National Family Health Survey-3
NMD: neuromuscular disorder
NMI-CP: neuromotor impairments including cerebral palsy
OAE: oto-acoustic emission
PAF: population attributable fraction
PDD-NOS: pervasive developmental disorder not otherwise specified
PPS: probability proportionate to population size
SC-ST: scheduled caste/tribe
SLI: Standard of Living Index
SQ: social quotient
STROBE: Strengthening the Reporting of Observational Studies in Epidemiology
TAG: Technical Advisory Group
TQ: Ten Questions
UHC: Universal Health Care
VI: vision impairment
VSMS: Vineland Social Maturity Scale
WMA: World Medical Association
