# Supplementary material for: Neurodevelopmental disorders in children aged 2–9 years: Population-based burden estimates across five regions in India
Source: PLoS Med. 2018 Jul 24;15(7):e1002615. doi: 10.1371/journal.pmed.1002615 (PMC6057634; doi:10.1371/journal.pmed.1002615)
Supplement: S1 STROBE Checklist — (DOCX) [file pmed.1002615.s001.docx]

| **S1 STROBE Checklist** | | | |
| --- | --- | --- | --- |
|  | **ITEM NUMBER** | **RECOMMENDATION** | **Sections** |
| TITLE AND ABSTRACT | 1 | (a) Indicate the study’s design with a commonly used term in the title or the abstract (b) Provide in the abstract an informative and balanced summary of what was done and what was found | (a) Title  (b) Abstract |
| **INTRODUCTION** |  |  |  |
| Background/ rationale | 2 | Explain the scientific background and rationale for the investigation being reported | Introduction |
| Objectives | 3 | State specific objectives, including any pre-specified hypotheses | Introduction: para 2 |
| **METHODS** |  |  |  |
| Study design | 4 | Present key elements of study design early in the paper | Methods: Para 2 (study design) |
| Setting | 5 | Describe the setting, locations, and relevant dates, including periods of recruitment, exposure, follow-up, and data collection | Methods:Para 4 (settings) |
| Participants | 6 | (a) Cohort study—Give the eligibility criteria, and the sources and methods of selection of participants. Describe methods of follow-up Case-control study—Give the eligibility criteria, and the sources and methods of case ascertainment and control selection. Give the rationale for the choice of cases and controls Cross-sectional study—Give the eligibility criteria, and the sources and methods of selection of participants (b) Cohort study—For matched studies, give matching criteria and number of exposed and unexposed Case-control study—For matched studies, give matching criteria and the number of controls per case | Methods: Para 5 (participants and recruitments) |
| Variables | 7 | Clearly define all outcomes, exposures, predictors, potential confounders, and effect modifiers. Give diagnostic criteria, if applicable | Methods: Para 8 (Study variables and measurements) |
| Data sources/ measurement | 8 | For each variable of interest, give sources of data and details of methods of assessment (measurement). Describe comparability of assessment methods if there is more than one group | Methods: Para 5, 8 (participants and recruitments and Study variables and measurements) |
| Bias | 9 | Describe any efforts to address potential sources of bias | Methods: Para 9 (sample size) and 10 (Analysis) |
| Study size | 10 | Explain how the study size was arrived at | Methods: Para 9 (sample size) |
| Quantitative variables | 11 | Explain how quantitative variables were handled in the analyses. If applicable, describe which groupings were chosen, and why | Methods: Para 8 (Study variables and measurements) and Para 10 (Analysis) |
| Statistical methods | 12 | (a) Describe all statistical methods, including those used to control for confounding (b) Describe any methods used to examine subgroups and interactions (c) Explain how missing data were addressed (d) Cohort study—If applicable, explain how loss to follow-up was addressed Case-control study—If applicable, explain how matching of cases and controls was addressed Cross-sectional study—If applicable, describe analytical methods taking account of sampling strategy (e) Describe any sensitivity analyses | Methods: Para 10 (Analysis) |
| **RESULTS** |  |  |  |
| Participants | 13 | (a) Report the numbers of individuals at each stage of the study—e.g., numbers potentially eligible, examined for eligibility, confirmed eligible, included in the study, completing follow-up, and analysed (b) Give reasons for non-participation at each stage (c) Consider use of a flow diagram | Results: Para 1 and 2 |
| Descriptive data | 14 | (a) Give characteristics of study participants (e.g., demographic, clinical, social) and information on exposures and potential confounders (b) Indicate the number of participants with missing data for each variable of interest (c) Cohort study—Summarise follow-up time (e.g., average and total amount) | Results: Para 2(Table 3) |
| Outcome data | 15 | Cohort study—Report numbers of outcome events or summary measures over time Case-control study—Report numbers in each exposure category, or summary measures of exposure Cross-sectional study—Report numbers of outcome events or summary measures | Results: Para 3(Prevalence of NDDs) |
| Main results | 16 | (a) Give unadjusted estimates and, if applicable, confounder-adjusted estimates and their precision (e.g., 95% confidence interval). Make clear which confounders were adjusted for and why they were included (b) Report category boundaries when continuous variables were categorized (c) If relevant, consider translating estimates of relative risk into absolute risk for a meaningful time period | Results: Para 3, 4 (Prevalence of NDDs) |
| Other analyses | 17 | Report other analyses done—e.g., analyses of subgroups and interactions, and sensitivity analyses | Results: Para 5 (Risk factor analysis) |

| **DISCUSSION** |  |  |  |
| --- | --- | --- | --- |
| Key results | 18 | summarize key results with reference to study objectives | Discussion: Para 1 |
| Limitations | 19 | Discuss limitations of the study, taking into account sources of potential bias or imprecision. Discuss both direction and magnitude of any potential bias | Discussion: Para 5 |
| Interpretation | 20 | Give a cautious overall interpretation of results considering objectives, limitations, multiplicity of analyses, results from similar studies, and other relevant evidence | Discussion: Para 6 |
| Generalizability | 21 | Discuss the generalizability (external validity) of the study results | Discussion: Para 6 |
| **OTHER INFORMATION** |  |  |  |
| Funding | 22 | Give the source of funding and the role of the funders for the present study and, if applicable, for the original study on which the present article is based | Funding: Para 1 |
