# Supplementary material for: Neurodevelopmental disorders in children aged 2–9 years: Population-based burden estimates across five regions in India
Source: PLoS Med. 2018 Jul 24;15(7):e1002615. doi: 10.1371/journal.pmed.1002615 (PMC6057634; doi:10.1371/journal.pmed.1002615)
Supplement: S1 Table — (DOCX) [file pmed.1002615.s002.docx]

| S1 Table. Comparison of “Drop-out” (N=181) and “Replacement” (N=158) participants in the study sites* | | | | | | | | |
| --- | --- | --- | --- | --- | --- | --- | --- | --- |
| Characteristic | **Palwal** | | **Kangra** | | **Dhenkanal** | | **Hyderabad** | |
|  | **Drop-out** | **Replacement** | **Drop-out** | **Replacement** | **Drop-out** | **Replacement** | **Drop-out** | **Replacement** |
| 2-<6 year Age Category |  |  |  |  |  |  |  |  |
| Age (months) (mean± std.dev.) | 45•6±13•6 | 45•7±13•3 | 50•7±13•1 | 46•4±13•9 | 43•2±11•2 | 42•4±11•3 | 38•3±11•6 | 45•6±12•9 |
| Weight (kg) (mean± std.dev.) | 14•3±4•6 | 13•4±2•7 | 12•9±2•3 | 13•2±2•8 | 63•2±67•8 | 47•3±59•1 | 12•1±2•3 | 13•4±2•8 |
| Height (cms) (mean± std.dev.) | 97•4±13•7 | 101•1±19•4 | 94•5±9•8 | 91±9•2 | 98•4±6•8 | 96•9±7•5 | 90•7±12•4 | 99•8±24•2 |
| Head circumference (cms) (mean± std.dev.) | 48•5±1•7 | 48•3±1•8 | 49•0±1•3 | 49•1±1•9 | 45•8±1•9 | 46•2±1•7 | 43•4±10•2 | 45•7±7•9 |
| Religion (% non-Hindu) | 28•6 | 14•8 | 11•8 | 29•4 | 2•9 | 4•0 | 21•4 | 28•6 |
| Caste (% SC-ST) | 25•9 | 40•7 | 18•8 | 15•4 | 23•5 | 44•0 | 23•1 | 41•7 |
| Mother as informant (%) | 28•6 | 40•7 | 76•5 | 88•2 | 88•6 | 96•0 | 92•9 | 78•6 |
| Rural residence (%) | 100•0 | 100•0 | 88•2 | 88•2 | 85•7 | 84•0 | 0•0 | 0•0 |
| 6-9 year Age Category |  |  |  |  |  |  |  |  |
| Age (months) (mean± std.dev.) | 86•1±8•0 | 87•6±12•1 | 91•4± 11•0 | 91•9± 13•9 | 87•6±10•1 | 87•9±8•0 | 89•8± 11•7 | 91•3± 11•3 |
| Weight (kg) (mean std.dev.) | 19•4±3•2 | 20•9±5•6 | 19•2± 3•4 | 18•3±3•4 | 62•9± 85•6 | 60•7± 78•9 | 29•4±41•4 | 41•9± 65•5 |
| Height (cms) (mean± std.dev.) | 118•5±6•1 | 121•1±11•4 | 114•1± 7•4 | 114•7± 9•9 | 121•1± 5•4 | 121•3± 4•5 | 116•8± 12•0 | 113•2± 19•8 |
| Head circumference (cms) (mean± std.dev.) | 49•7±1•9 | 50±1•9 | 50•1±2•3 | 50•6±1•8 | 50•8± 2•3 | 51•0± 2•4 | 47•4± 6•7 | 47•4± 6•7 |
| Religion (% non-Hindu) | 27•8 | 23•5 | 15•0 | 15•0 | 3•5 | 5•0 | 35•0 | 27•8 |
| Caste (% SC-ST) | 38•9 | 31•3 | 10•5 | 22•2 | 39•3 | 21•1 | 23•5 | 12•5 |
| Mother as informant (%) | 33•3 | 41•2 | 95•0 | 100•0 | 96•6 | 95•0 | 80•0 | 33•3 |
| Rural residence (%) | 100•0 | 100•0 | 75•0 | 100•0 | 89•7 | 95•0 | 0•0 | 0•0 |
| **Drop-outs were replaced with age category and sex matched children; Goa did not have any drop-out* | | | | | | | |  |
