# Supplementary material for: Neurodevelopmental disorders in children aged 2–9 years: Population-based burden estimates across five regions in India
Source: PLoS Med. 2018 Jul 24;15(7):e1002615. doi: 10.1371/journal.pmed.1002615 (PMC6057634; doi:10.1371/journal.pmed.1002615)
Supplement: S2 Table — NDD, neurodevelopmental disorder. (DOCX) [file pmed.1002615.s003.docx]

| S2 Table. Co-existing Neuro-developmental Disorders (NDD; %) in study participants with at least one NDD | | | | | | | | | | | |
| --- | --- | --- | --- | --- | --- | --- | --- | --- | --- | --- | --- |
| Children with NDD (N=475) | **Vision Impairment** | **Epilepsy** | **Neuromotor Impairments including Cerebral Palsy** | **Hearing Impairment** | **Speech & Language Disorders** | **ASD** | **Intellectual Disability** | **ADHD Disorder** | **Learning Disabilities** | **Overall* (at least 1 co–existent NDD)** | |
| Vision impairment (n=22) | – | 18•2 | 22•7 | 9•1 | 4•6 | 13•6 | 27•3 | 0•0 | NA | 40•9 | |
| Epilepsy (n=69) | 5•8 | – | 26•1 | 8•7 | 4•4 | 17•4 | 40•6 | 1•5 | 1•5 | 55•1 | |
| Neuro-motor Impairments including Cerebral Palsy (n=62) | 8•1 | 29•0 | – | 6•5 | 4•8 | 16•1 | 66•1 | 0•0 | 0•0 | 74•2 | |
| Hearing Impairment (n=136) | 1•5 | 4•4 | 2•9 | – | NA | 2•9 | 12•5 | 0•0 | NA | 16•2 | |
| Speech and Language Disorders (n=83) | 1•2 | 3•6 | 3•6 | NA | – | NA | NA | 3•6 | 2•4 | 13•3 | |
| Autism Spectrum Disorders (ASD) (n=44) | 6•8 | 27•3 | 22•7 | 9•1 | NA | – | 72•7 | NA | 2•3 | 79•6 | |
| Intellectual Disability (n=144) | 4•2 | 19•4 | 28•5 | 11•8 | NA | 22•2 | – | NA | NA | 56•9 | |
| Attention Deficit Hyperactivity Disorder (n=27) | 0•0 | 3•7 | 0•0 | 0•0 | 11•1 | NA | NA | – | 0•0 | 14•8 | |
| Learning Disabilities (n=36) | NA | 2•8 | 0•0 | NA | 5•6 | 2•8 | NA | 0•0 | – | 11•1 | |
| ** Of 475 children with NDDs, 103 (21*•*7%) children had >1 NDDs; 70 (14*•*7%), 23 (4*•*8%), 8 (1*•*7%), 2 (0*•*4%) had two, three, four and five coexisting NDDs respectively.NA: Not applicable; excluded as per necessary preconditions for diagnosis* | | | | | | | | | | |  |
