# Supplementary material for: Neurodevelopmental disorders in children aged 2–9 years: Population-based burden estimates across five regions in India
Source: PLoS Med. 2018 Jul 24;15(7):e1002615. doi: 10.1371/journal.pmed.1002615 (PMC6057634; doi:10.1371/journal.pmed.1002615)
Supplement: S3 Table — NDD, neurodevelopmental disorder. (DOCX) [file pmed.1002615.s004.docx]

| S3 Table. Distribution of the risk factors among children with and without Neuro-developmental Disorders (NDD)^#^ | | | | |
| --- | --- | --- | --- | --- |
| Risk factors | **Distribution of risk factors Estimated%(95%CI)** | | **P value** | |
|  | **Children with NDD** | **Children without NDD** |  |  |
| Modifiable Risk factors |  |  |  | |
| Consanguinity | 7•7 (5•0- 11•7) | 7•8 (6•2- 9•7) | 0•968 | |
| Neurological or mental illness in family | 10•0 (7•4- 13•5) | 9•9 (8•3- 11•7) | 0•918 | |
| Previous pregnancy loss in the mother | 18•5 (15•0- 22•5) | 16•8 (14•9- 18•7) | 0•359 | |
| Medical disorders of pregnancy | 12•4 (8•4- 17•9) | 9•5 (7•9- 11•4) | 0•203 | |
| Medication during pregnancy | 34•6 (26•3- 43•9) | 27•4 (22•3- 33•0) | 0•063 | |
| Radiation exposure | 3•4 (1•3- 9•1) | 6•1 (3•4- 10•6) | 0•265 | |
| Pesticide exposure | 0•1 (0•0- 1•0) | 0•3 (0•0- 1•1) | 0•528 | |
| Chorioamnionitis | 5•6 (3•0- 10•1) | 3•1 (2•3- 4•2) | 0•075 | |
| Birth order ≥3* | 25•7 (19•3- 33•4) | 15•9 (13•9- 18•1) | 0•001* | |
| Multiple pregnancies | 5•2 (2•4- 10•8) | 6•4 (4•4- 9•1) | 0•611 | |
| Home/unattended delivery | 36•8 (29•4- 44•9) | 23•9 (20•8- 27•3) | <0•001* | |
| Emergency caesarean section/instrumental delivery | 18•3 (12•7- 25•6) | 15•2 (12•8- 18•0) | 0•326 | |
| Perinatal asphyxia* | 14•5 (9•4- 21•6) | 6•2 (4•8- 8•0) | <0•001* | |
| Neonatal illness with or without altered sensorium | 14•6 (11•0- 19•1) | 6•8 (5•6- 8•4) | <0•001* | |
| Traumatic brain injury | 5•7 (3•8- 8•6) | 3•0 (2•0- 4•4) | 0•020* | |
| Post natal neurological infections | 3•5 (2•1- 5•6) | 0•7 (0•4- 1•4) | <0•001* | |
| Stunting | 39•0 (31•5- 47•0) | 26•6 (23•9- 29•5) | 0•002* | |
| Underweight | 38•6 (32•3- 45•4) | 27•2 (24•6- 30•0) | <0•001* | |
| Low birth weight (<2•5 kg) or prematurity(gestation<37 weeks) | 15•4 (11•3- 20•7) | 11•4 (9•9- 13•1) | 0•094 | |
| Non modifiable risk factors |  |  |  | |
| Gender (Boy) | 53•9 (47•1- 60•6) | 48•4 (46•4- 50•4) | 0•146 | |
| Place of Residence (Rural) | 51•3 (43•6- 59•0) | 45•2(39•8- 50•7) | 0•085 | |
| Education (never been to school) | 35•1 (27•9- 43•1) | 24•9 (21•9- 28•1) | 0•007* | |
| Religion (non-Hindu) | 34•7 (26•2- 44•1) | 33•1 (27•8- 39•0) | 0•727 | |
| Caste (Scheduled Caste or Tribe) | 27•8 (22•3- 34•1) | 25•7 (22•4- 29•3) | 0•547 | |
| Age category(6-9 years) | 57•2 (49•4- 64•8) | 46•3 (40•3- 52.4) | 0•004* | |
| *# Weighed according to national population for age category, gender,place of residence (rural/ urban) and religion (Hindu/ non-Hindu) as per Census of India, 2011;* Statistically significant (p<0.05)* | | | |  |
