# Supplementary material for: Neurodevelopmental disorders in children aged 2–9 years: Population-based burden estimates across five regions in India
Source: PLoS Med. 2018 Jul 24;15(7):e1002615. doi: 10.1371/journal.pmed.1002615 (PMC6057634; doi:10.1371/journal.pmed.1002615)
Supplement: S4 Table — NDD, neurodevelopmental disorder. (DOCX) [file pmed.1002615.s005.docx]

| S4 Table. Multi-variable logistic regression analysis for risk factors for specific NDDs in children aged 2-9 years | | | | | | | | | | | | | | | |
| --- | --- | --- | --- | --- | --- | --- | --- | --- | --- | --- | --- | --- | --- | --- | --- |
| Risk factors | **Vision Impairment** | | **Epilepsy** | | **Neuro-motor Impairments including Cerebral Palsy** | | **Hearing Impairment** | | **Speech and Language Disorders** | | **Autism Spectrum Disorders** | | | **Intellectual Disability** | |
| Modifiable Risk factors | **AOR** | **p value** | **AOR** | **p value** | **AOR** | **p value** | **AOR** | **p value** | **AOR** | **p value** | **AOR** | | **p value** | **AOR** | **p value** |
| Neurological or mental illness in the family | 0•5 (0•1- 2•6) | 0•433 | 1•8 (0•9- 3•8) | 0•119 | 0•8 (0•2- 2•7) | 0•694 | 1•4 (0•7- 2•7) | 0•327 | 0•4 (0•1- 1•0) | **0•050** | 1•7 (0•7- 4•1) | | 0•264 | 1•7 (0•9- 3•4) | 0•103 |
| Medical complications during pregnancy | 1•0 (0•2- 5•9) | 0•958 | 0•7 (0•3- 1•6) | 0•400 | 0•3 (0•1- 1•1) | 0•077 | 0•9 (0•4- 1•9) | 0•779 | 2•0 (1•0- 3•8) | **0•044** | 0•8 (0•3- 2•5) | | 0•768 | 0•7 (0•3- 1•5) | 0•323 |
| Chorioamnionitis | 1•7 (0•1- 20•1) | 0•692 | 0•7 (0•2- 2•6) | 0•585 | 0•7 (0•2- 2•8) | 0•584 | 2•1 (1•0- 4•3) | **0•055** | 3•0 (1•1- 8•4) | **0•036** | 2•0 (0•6- 7•3) | | 0•272 | 1•1 (0•4- 2•7) | 0•833 |
| Birth order ≥3 | 0•8 (0•1- 3•2) | 0•701 | 0•6 (0•3- 1•2) | 0•148 | 1•0 (0•5- 2•4) | 0•910 | 1•6 (1•0- 2•5) | **0•029** | 0•5 (0•2- 1•3) | 0•150 | 1•0 (0•3- 3•5) | | 0•988 | 1•3 (0•7- 2•5) | 0•423 |
| Multiple pregnancies | - | - | 0•5 (0•1- 3•0) | 0•407 | 0•3 (0•1- 1•5) | 0•130 | 1•4 (0•4- 4•6) | 0•627 | 0•5 (0•1- 2•0) | 0•351 | 0•2 (0•02- 3•3) | | 0•285 | 0•8 (0•3- 2•3) | 0•651 |
| Place of delivery (Non-institutional) | 0•6 (0•2- 2•4) | 0•487 | 0•7 (0•3- 1•9) | 0•527 | 1•4 (0•4- 4•3) | 0•601 | 2•4 (1•5- 3•9) | **<0•001** | 1•8 (0•9- 3•7) | 0•083 | 0•5 (0•2- 1•4) | | 0•194 | 1•2 (0•7- 2•1) | 0•544 |
| Perinatal asphyxia | 3•6 (1•2- 11•4) | **0•027** | 2•3 (0•9- 5•9) | 0•090 | 7•6 (2•9- 20•4) | **<0•001** | 0•6 (0•3- 1•4) | 0•222 | 0•3 (0•1- 1•3) | 0•099 | 3•9 (1•1- 13•9) | | **0•033** | 2•1 (1•1- 4•0) | **0•023** |
| Neonatal illness | 0•3 (0•1- 1•1) | 0•075 | 3•2 (1•5- 7•0) | **0•004** | 3•6 (1•4- 9•3) | **0•009** | 1•7 (0•9- 3•2) | 0•084 | 1•5 (0•7- 3•2) | 0•250 | 1•0 (0•2- 4•8) | | 0•957 | 2•6 (1•5- 4•5) | **0•001** |
| Traumatic brain injury | - | - | 1•7 (0•4- 6•5) | 0•466 | 1•0 (0•1- 8•7) | 0•977 | 2•1 (0•7- 5•9) | 0•163 | 1•3 (0•4- 4•9) | 0•685 | 1•0 (0•2- 5•8) | | 0•978 | 1•9 (0•7- 5•2) | 0•194 |
| Post-natal neurological/brain infections | 8•4 (2•3- 30•9) | **0•002** | 2•7 (0•7- 10•3) | 0•145 | 8•3 (1•4- 50•4) | **0•022** | 1•4 (0•5- 3•8) | 0•541 | 0•7 (0•1- 7•2) | 0•778 | 2•2 (0•2- 27•1) | | 0•545 | 7•1 (2•6- 19•5) | **<0•001** |
| Stunting | 1•0 (0•3- 2•9) | 0•929 | 4•4 (1•8- 10•8) | **0•001** | 9•0 (3•2- 25•6) | **<0•001** | 1•3 (0•8- 2•3) | 0•329 | 1•0 (0•6- 1•8) | 0•987 | 2•7 (0•6- 11•3) | | 0•171 | 3•9 (2•1- 7•3) | **<0•001** |
| Low birth weight(<2•5kg)/ prematurity(gestation <37weeks) | 2•1 (0•7- 6•5) | 0•187 | 1•0 (0•5- 2•2) | 0•968 | 3•7 (1•6- 8•4) | **0•002** | 1•6 (0•9- 2•8) | 0•113 | 2•2 (1•2- 4•3) | **0•015** | 1•6 (0•5- 4•9) | | 0•449 | 1•7 (1•0- 3•0) | **0•048** |
| Non-modifiable risk factors | | | | | | | | | | | | |  |  |  |
| Gender (Boy) | 1•4 (0•5- 4•3) | 0•528 | 2•4 (1•2- 4•8) | **0•018** | 1•4 (0•6- 3•1) | 0•445 | 1•1 (0•7- 1•8) | 0•675 | 1•2 (0•7- 2•2) | 0•429 | | 1•7 (0•6- 4•7) | 0•341 | 1•0 (0•6- 1•6) | 0•930 |
| Place of Residence (Rural) | 2•9 (0•4- 24•0) | 0•313 | 1•2 (0•6- 2•6) | 0•622 | 0•7 (0•2- 1•8) | 0•414 | 5•1 (1•9- 13•4) | **0•001** | 1•0 (0•4- 2•2) | 0•935 | | 0•8 (0•3- 2•7) | 0•775 | 1•0 (0•5- 1•7) | 0•887 |
| Education | 0•7 (0•1- 3•8) | 0•708 | 1•9 (0•5- 7•0) | 0•307 | 1•4 (0•3- 7•6) | 0•657 | 1•5 (0•9- 2•3) | 0•117 | 0•6 (0•2- 1•5) | 0•245 | | 2•0 (0•3- 13•0) | 0•454 | 1•7 (0•7- 3•9) | 0•236 |
| Religion (non-Hindu) | - | - | 0•9 (0•3- 3•0) | 0•873 | 2•3 (0•8- 6•1) | 0•107 | 0•8 (0•4- 1•5) | 0•465 | 0•6 (0•2- 1•5) | 0•279 | | 1•2 (0•3- 4•6) | 0•817 | 0•5 (0•2- 1•4) | 0•200 |
| Caste (Scheduled Caste or Tribe) | 0•9 (0•3- 2•4) | 0•853 | 1•3 (0•6- 2•9) | 0•550 | 1•1 (0•4- 2•9) | 0•908 | 1•1 (0•6- 2•0) | 0•700 | 0•8 (0•4- 1•6) | 0•279 | | 0•4 (0•1- 1•0) | 0•204 | 0•7 (0•4- 1•3) | 0•289 |
| Standard of Living Index Score | 0•98 (0•92- 1•04) | 0•479 | 1•0 (0•6- 2•9) | 0•390 | 0•99 (0•95- 1•03) | 0•681 | 0•98 (0•96- 1•00) | 0•077 | 0•99 (0•96- 1•02) | 0•538 | | 0•99 (0•94- 1•04) | 0•633 | 0•98 (0•95- 1•01) | 0•192 |
| Age category (6-9 years) | 1•1 (0•4- 3•1) | 0•909 | 3•3 (1•4- 7•7) | **0•006** | 0•99 (0•98- 1•01) | 0•830 | 0•9 (0•6- 1•4) | 0•687 | 0•8 (0•4- 1•6) | 0•574 | | 2•0 (0•6- 6•5) | 0•268 | 1•7 (0•9- 3•0) | 0•076 |
| *Weighed according to national population for age category, sex, place of residence (rural/urban) and religion (Hindu/ non-Hindu) (Census of India, 2011);*  *Highlighted are statistically significant; **calculated for significant modifiable factors; AOR: adjusted odds ratio; Values in parentheses indicate 95% confidence interval* | | | | | | | | | | | | | | |  |
