# Supplementary material for: Neurodevelopmental disorders in children aged 2–9 years: Population-based burden estimates across five regions in India
Source: PLoS Med. 2018 Jul 24;15(7):e1002615. doi: 10.1371/journal.pmed.1002615 (PMC6057634; doi:10.1371/journal.pmed.1002615)
Supplement: S5 Table — (DOCX) [file pmed.1002615.s006.docx]

| S5 Table. Comparison of demographic features (%) of the participant population with National databases | | | |
| --- | --- | --- | --- |
| Attribute | **India** | | **Study (95% CI)** |
| Gender: % Female (2-9 year age group) | 47•9 [1] | | 49•4 (47•8-51•0) |
| Residence: % Rural (2-9 year age group) | 74•0 [1] | | 79•0 (77•7-80•3) |
| Religion: % Non-Hindu (2-9 year age group) | 22•3 [1] | | 10•0 (9•1-11•0) |
| Caste (2-9 year age group) |  |  | |
| *% Scheduled Caste/Tribe* | 27•9 [1] | | 28•0 (26•6-29•5) |
| Socio-Economic Status (Standard of Living) [2] |  | |  |
| *Lower* | 22•8 | | 14•2 (13•1-15•4) |
| *Lower middle* | 20•0 | | 13•0 (11•9-14•1) |
| *Middle* | 18•3 | | 21•0 (19•6-22•3) |
| *Upper middle* | 21•8 | | 33•0 (31•4-34•6) |
| *Upper* | 17•6 | | 19•0 (17•6-20•3) |
| Education |  | |  |
| *% Educated <5 Year (ever married)* | 44•3 [1] | | 42•3 (40•7-43•9)  (respondents) |
| *% Children (6-9 years) currently in school* | 95•0 [3] | | 95•4 (94•4-96•3) |
| Maternal, Newborn, Child Health and Nutrition |  | |  |
| *% Stunted (under 5 years)* | 38•4 [3] | | 29•5 (27•5-31•5) |
| *% Delivered at Home* | 33•0 [1] | | 33•3 (31•8-34•9) |
| *% Low birth Weight (IUGR/ Pre-term)* | 18•0 [3] | | 12•5(11•4-13•6) |

**References**

Census of India. Office of the Registrar General and Census Commissioner of India, Ministry of Home Affairs, Government of India, 2011.

1. International Institute for Population Sciences (IIPS) and Macro International. National Family Health Survey (NFHS-3), 2005–06: India: Volume I. Mumbai: IIPS.
2. International Institute for Population Sciences (IIPS) and ICF. 2017. National Family Health Survey (NFHS-4), 2015-16: India. Mumbai: IIPS.
