# Supplementary material for: Neurodevelopmental disorders in children aged 2–9 years: Population-based burden estimates across five regions in India
Source: PLoS Med. 2018 Jul 24;15(7):e1002615. doi: 10.1371/journal.pmed.1002615 (PMC6057634; doi:10.1371/journal.pmed.1002615)
Supplement: S6 Table — (DOCX) [file pmed.1002615.s007.docx]

| S6 Table. Summary description of four diagnostic tools developed and validated by INCLEN team for the study | | |
| --- | --- | --- |
| NDD assessed | **Diagnostic Instrument** | **Brief description about the tool** |
| Epilepsy | INCLEN Diagnostic Tool for Epilepsy (INDT–EPI) [1] | ***Tool:*** INDT-EPI included questions in simple language to elicit the history of common seizure types (generalized and partial motor seizures, absence seizures and myoclonic seizures) from the primary care provider for the child. The tool also elicits the number of seizures and duration between first and last seizures, provoked seizures such as febrile seizures, seizures occurring during neuro-infections, with head trauma or during systemic illnesses and if the seizure mimics events such as breath holding spells and syncopal attacks. Last question is on final diagnosis. |
|  |  | ***Sensitivity and Specificity:*** Children aged 2-9 years, enrolled by systematic random sampling at paediatric neurology out-patient clinics of three tertiary care centres were independently evaluated in a blinded manner by primary care physicians trained to administer the test, and by teams of two paediatric neurologists.The test for epilepsy had sensitivity and specificity of 85•8% and 95•3%; positive and negative predictive values of 94•0% and 88•5%; and positive and negative likelihood ratios of 18•25 and 0•15, respectively. |
| Neuro-motor Impairments including Cerebral Palsy (NMI–CP) | INCLEN Diagnostic Tool for Neuro–Motor Impairment (INDT–NMI) [2] | ***Tool:* The INDT-NMI developed comprises of three sections**.   - Section-I (Triage questions) consists of four questions to elicit information from the parents/primary caregiver of the child regarding attainment of selected motor developmental milestones. - Section*-*II (Observations): Physician makes three observations for assessing hand function, gait and muscle weakness. Section*-*III consists of six questions, and the operator (graduate physician) does the neurological examination necessary for confirmation of NMI.Thefinal diagnosis of NMI-CP is derived through an algorithm based on interpretation of three sections (*i.e.*13 questions/items) and information on age at onset of symptoms, course of the illness and obvious clinical evidence of involvement of spinal cord (*i.e.* pilonidal sinus, tuft of hair). |
|  |  | ***Sensitivity and Specificity:*** Children aged 2-9 years selected through systematic random sampling, underwent assessment for identification and classification of neuro-motor impairments (NMI).The overall sensitivity of the INDTNMI was 75•4% and specificity was 86•8%. INDT-NMI helped graduate physicians to correctly classify 86•6% (112/129) children with NMI into different types (cerebral palsy, neuro-motor diseases and other NMI).Graduate physicians assigned 40 children (8•8%) as ‘indeterminate’, 38 (95%) of whom had either NDD and/or NMI and thus merited referral. |
| Autism Spectrum Disorders (ASD) | INCLEN Diagnostic Tool for Autism Spectrum Disorders (INDT–ASD) [3] | ***Tool:*** The tool has two sections: Section A has 29 symptoms/items and Section B contains 12 questions corresponding to B and C domains of DSM-IV-TR, time of onset, duration of symptoms, score and diagnostic algorithm trichotomous endorsement choice (‘yes’, ‘no’, ‘unsure/not applicable’) is given to the assessor/ interviewer. In addition, the clinician/psychologist has to make behavioral Observations on the child and score the item as well.For any discrepancy in parental response and interviewer’s assessment, it is indicated for each question whether parental response or assessor’s observation should take precedence. Each symptom/item is given a score of ‘1’ for ‘Yes’ and ‘0’ for ‘No’ or ‘unsure/not applicable’. Presence of ≥ 6 symptoms/item (or score of ≥ 6), with at least two symptom/item each from impaired communication and restricted repetitive pattern of behavior, is used to diagnose ASD |
|  |  | ***Sensitivity and Specificity:*** INDT-ASD and Childhood Autism Rating Scale (CARS) were administered in a randomly decided sequence by trained psychologist, followed by an expert evaluation by DSM-IV TR diagnostic criteria (gold standard).The overall diagnostic accuracy (AUC=0•97, 95% CI 0•93, 0•99; *P*<0•001) and validity (sensitivity 98%, specificity 95%, positive predictive value 91%, negative predictive value 99%) of INDT-ASD for Autism spectrum disorder were high, taking expert diagnosis using DSM-IV-TR as gold standard. |
| Attention Deficit Hyperactivity Disorder (ADHD) | INCLEN Diagnostic Tool for Attention Deficit Hyperactivity Disorder (INDT–ADHD) [4] | ***Tool:*** INDT-ADHD consists of 18 items related to ‘inattention’ and ‘hyperactivity/impulsiveness’ symptoms (9 items each) while Section B consists of 8 items pertaining to onset, duration, functional impairment and a diagnostic algorithm to arrive at the diagnosis. Scoring is by parental endorsement with ‘1’ for ‘Yes’ and ‘0’ for ‘No’. A score of six or more of the 9 items related to ‘only inattention’, ‘only ‘hyperactivity/ impulsiveness’ and ‘both’ indicate ‘predominantly inattentive’, ‘predominantly hyperactive/impulsive’, and ‘combined subtypes’, respectively. These are considered significant if the duration of symptom is ≥6 months, onset is before 7 years of age, and manifestation are in at least two settings. |
|  |  | ***Sensitivity and Specificity:*** INDT-ADHD and Connor’s 3 Parent Rating Scale (C3PS) were administered, followed by an expert evaluation by DSM-IV-TR diagnostic criteria. Psychometric parameters of INDT-ADHD for differentiating attention deficit hyperactivity disorder and normal children were: sensitivity 87•7%, specificity 97•2%, positive predictive value 98•0% and negative predictive value 83•3%, whereas for differentiating from other neuro-developmental disorders were 87•7%, 42•9%, 58•1% and 79•4%, respectively. |

**References**

1. Konanki R, Mishra D, Gulati S, Aneja S, Deshmukh V, Silberberg D, et al. INCLEN Diagnostic Tool for Epilepsy (INDT-EPI) for Primary Care Physicians: Development and Validation. Indian Pediatr 2014; 51: 539-543.
2. Gulati S, Aneja S, Juneja M, Mukherjee S, Deshmukh V, Silberberg, et al. INCLEN Diagnostic Tool for Neuro-motor Impairment (INDT-NMI) for Primary Care Physician: Development and Validation. Indian Pediatr 2014; 51: 613-619.
3. Juneja M, Mishra D, Russell PS, Gulati S, Deshmukh V, Tudu P, et al. INCLEN Diagnostic Tool for Autism Spectrum Disorder (INDT-ASD): Development and Validation. Indian Paediatr 2014; 51: 359-365.
4. Mukherjee S, Aneja S, Russell PS, Gulati S, Deshmukh V, Sagar R, et al. INCLEN Diagnostic Tool for Attention Deficit Hyperactivity Disorder (INDT-ADHD): Development and Validation. Indian Paediatr 2014; 51: 457-62.
