# Supplementary material for: Neurodevelopmental disorders in children aged 2–9 years: Population-based burden estimates across five regions in India
Source: PLoS Med. 2018 Jul 24;15(7):e1002615. doi: 10.1371/journal.pmed.1002615 (PMC6057634; doi:10.1371/journal.pmed.1002615)
Supplement: S1 Text — (DOCX) [file pmed.1002615.s008.docx]

**S1 Text. Prospectively written statistical analysis plan**

***Analysis:***  Before proceeding with the analysis of the data collected to assess the screening and diagnostic prevalence of neuro-developmental disabilities, a summary of each variable, or group of variables, will be produced.  Graphical methods including histograms, scatter plots, and box plots, will be used at this stage in order to understand aspects of the distribution of variables and the quality of data. Survey commands will be used to analyze the data giving due weight to rural and urban population of the zone as per 2001 census. Screening prevalence and the diagnostic prevalence of specific disabilities will be calculated. Descriptive analyses will be carried out to compare the outcomes by zones (e.g., geopolitical divisions).Means, standard deviations, medians, and ranges will be computed for measured continuous variables; marginal distributions will be used for categorical/ binary data. Confidence intervals (95%) along with design effect (Deff) will be used to give the reader a sense of underlying variations in study results due to random error and clustering.

To address the aim of exploring the role of modifiable risk factors, the case (developmental disability) versus control distribution of these potential risk factors will be compared by chi‑square tests or Fisher's exact tests, wherever appropriate. Case and control comparisons on the basis of continuous factors will use traditional methods for summarization and Student's t‑test analysis and ANOVA to test for statistical significance. We will use the odds ratio (OR) as the basic summary statistic to assess alterations in risk. Initially, we will estimate crude odds ratios (cOR). Adjusted OR’s will then be computed from the stratified and logistic regression analyses; discussions and decisions on the analytic strategies as well as the statistical hypothesis tests will be done at the planned workshops.

**Reference**

Grant Application “Neuro-developmental Disabilities among Children in India: An INCLEN Study” (NIH 1R21-HD53057-01A1)
